# Association of maternal hypertensive disorders with retinopathy of prematurity: A systematic review and meta-analysis

**DOI:** 10.1371/journal.pone.0175374

**Published:** 2017-04-07

**Authors:** TingTing Zhu, Li Zhang, FengYan Zhao, Yi Qu, Dezhi Mu

**Affiliations:** 1 Department of Pediatrics, West China Second University Hospital, Sichuan University, Chengdu, China; 2 Key Laboratory of Obstetric & Gynecologic and Pediatric Diseases and Birth Defects of Ministry of Education, Sichuan University, Chengdu, Sichuan, China; 3 Department of Pediatrics and Neurology, University of California, San Francisco, San Francisco, California, United States of America; Centre Hospitalier Universitaire Vaudois, FRANCE

## Abstract

**Backgroud:**

The role of maternal hypertensive disorders in pregnancy (HDP) in the development of retinopathy of prematurity (ROP) is unclear.

**Methods:**

Studies were retrieved through literature searches in PubMed, EMBASE, Web of Science and the Cochrane Library up to May 5, 2016 without language restrictions. Cohort or case–control studies that reported the association of maternal hypertensive disorders and retinopathy of prematurity were eligible. Either a fixed- or a random-effects model was used to calculate the overall combined risk estimates.

**Results:**

Thirteen cohort studies involving a total of 45082 individuals were included in the review. The pooled odds ratios of maternal hypertensive disorders in pregnancy for any stage and severe stages of ROP was 1.12 (95%CI: 0.90–1.40) and 0.80 (95%CI: 0.47–1.35), respectively. Sensitivity analyses confirmed that no single study qualitatively influenced the pooled OR. However, substantial heterogeneity and publication bias were observed in the meta-analysis.

**Conclusions:**

Additional larger, prospective and well-adjusted studies are needed to determine the association between HDP and ROP, especially regarding the effects of different types of maternal hypertensive disorders in pregnancy on retinopathy of prematurity.

## Introduction

Retinopathy of prematurity (ROP), a multiple-factor-induced disease of abnormal retinal vascular proliferation, often occurs in premature infants and low-birth-weight infants and may lead to blindness. With advances in neonatal care, increasing survival of premature infants has been achieved despite increased incidence of ROP. ROP has remained one of the leading causes of preventable childhood blindness, and occurs frequently in middle-income countries such as Latin America and some eastern European countries [1]. Also Preterm was found to be associated visual impairment and estimates of retinopathy of prematurity at regional and global levels for 2010 [2]. In addition to blindness, ROP can lead to eye or visual problems, such as visual impairment, strabismus, or major refractive error [3]. Infants with ROP have been found to be at risk of motor impairment, cognitive impairment, and severe hearing loss [4]. ROP pathogenesis has evolved over the past 50 years, yet the etiology is not fully understood. Some studies have proposed that antenatal or maternal risk factors might lead to ROP [5].

Hypertensive disorders in pregnancy (HDP) comprise a wide spectrum of disorders before or during pregnancy, which are generally characterized by a blood pressure above 140/90 mm Hg recorded with or without edema and proteinuria [6]. These disorders were clinically classified as chronic hypertension, gestational hypertension, preeclampsia, eclampsia, preeclampsia superimposed on chronic hypertension, and unspecified hypertension [7]. In total, 6% to 8% of pregnant women suffer from this complication [8]. Preeclampsia is characterized by the occurrence of hypertension and/or proteinuria after 20 weeks of gestation. The presence of HDP influences the intrauterine environment and contributes significantly to maternal and perinatal morbidity and mortality. [8–9]. In 1996, Gerd et al first described the relationship between preeclampsia and ROP [10]. Since then, opposing results have been observed in a series of studies, making the association of HDP with ROP inconclusive. Determining the association between HDP and ROP may have better understanding of ROP and clinical implications given the possibility that prevention and treatment of HDP might reduce the incidence of ROP. Thus, the objective of this systematic review is to determine the impact of HDP on ROP using cohort and case-control studies that assess the association between HDP and ROP published before 2016.

## Methods

This article was followed the PRISMA and MOOSE Guidelines for meta-analysis.

### Literature search

The PubMed, EMBASE, Web of Science and Cochrane Library databases were searched from initial to May 5 2016. Search terms consisted of a combination of Medical Subject Headings ("Pregnancy Complications" “Pre-Eclampsia”, “retinopathy of prematurity”) and key words (“maternal hypertension”, “gestational hypertension”, “retinopathy of prematurity”) without restriction to language. Potential articles were identified by reading titles and abstracts. If the titles and abstracts suggested that the study discussed risk factors for ROP, then two authors (TTZ and YQ) independently read the full text of the studies and decided whether the studies met the inclusion criteria. Disagreements were resolved by a third author (ZFY), who independently examined the studies, and a consensus was then reached. The reference lists of the included studies and relevant reviews were hand searched for further relevant articles.

### Study selection

Original studies evaluating the association between ROP and HDP were included if: (i) case-control study or cohort study; (ii) The diagnosis of ROP was based on the results of ophthalmoscopy. The stage from 1 to 5 of ROP was classified according to the International Classification of ROP [11]. Stage 3, more than stage 3 and need for surgery for ROP were deemed to be severe ROP; (iii) The study involved human subjects; (iv) provided relative risk (RR) estimates such as risk ratios, incidence rate ratios, hazard ratios or odds ratios (OR) and 95%CIs (CIs) for HDP, or raw data from which these factors could be calculated. Two authors independently examined the studies for eligibility. Review articles, commentary articles, animal studies, letters, and case-series were excluded.

### Data extraction

Using a form of data extraction, two authors independently collected data. The results were compared between collectors, and any uncertainties were resolved by consensus with a third author. The following characteristics of the study were recorded during data extraction: first author, year of publication, country, study design, inclusion population, number of study, gestational age, birth weight, diagnosis of ROP, impact of HDP on ROP, and confounders adjusted for. Studies without these information were excluded. We included the study with the largest number of participants if populations overlapped between studies.

### Quality assessment and risk of bias

To assess the risk bias of the studies, two authors screened the study design, the size and representativeness of the study population, the validity of outcomes, and the quality of the statistical analysis. The Newcastle-Ottawa Quality Assessment Scale (NOS) was used [12]. This scale is widely applied to evaluate case-control or cohort studies with maximum scores of nine stars, which comprises eight items such as selection bias, comparability bias, exposure bias and outcome bias. Studies possessing five or more stars were deemed to be high quality studies. Any disagreement was settled as described above.

### Statistical analysis

For the included studies, OR was used to evaluate the association between HDP and ROP. For one study that reported ORs separately for preeclampsia and gestational hypertension, we combined these 2 groups into a single group and calculated a combined OR using a fixed-effects model for the main analysis. Effect measures were weighted by log inverse variance. Combined results were performed by using random-effects or fixed-effects models. Heterogeneity was examined by the Q statistic (P< 0.1 was considered to represent significant) and the I^2^ test (values of > 50% was considered to represent significant). If significant heterogeneity was observed between the studies, the pooled OR was estimated using a random effects model. Otherwise, a fixed effects model was used. To assess the stability of the results, a sensitivity analysis was performed by removing each individual study in turn from the total, and re-analyzing the remaining studies. To find the source of the heterogeneity, we performed subgroup analyses combined with meta-regression according to the variance in the studies. Publication bias was evaluated by visual funnel plots and Begg's linear regression test. All statistical analyses were performed using Review Manager 5.3 and Stata 12.0.

## Results

### Search results and the characteristics of the included studies

**Fig 1:**
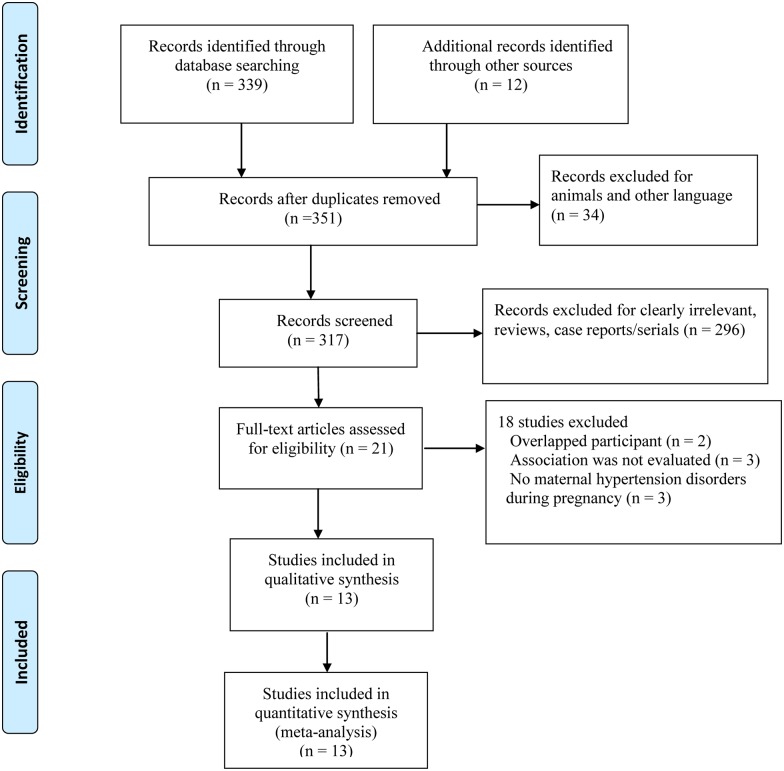
Flow diagram of literature screen process.

The literature search identified 351 studies based on our search strategy, of which, thirteen cohort studies with a total of 45082 babies were selected for analysis (Fig 1). Characteristics of included studies are summarized in Table 1 [5, 8–10, 13–21]. These studies were published between 1996 and 2016; two of them were conducted in the United States, three in Turkey, two in Italy, one in Germany, one in Sweden, two in China, one in Singapore, and one in Brazil. All participants were preterm or very low birth weight infants. For diagnosis of ROP, included studies measured or used register data based on ophthalmoscopy. The category of HDP was consistent among studies. Ten studies reported preeclampsia [5,10,13–18,20–21], two reported gestational hypertension [16,20], and two reported HDP [8–9]. Outcomes were categorized as 2 broad categories: “any stage ROP” and “severe ROP”.

**Table 1:** Characteristics of included studies.

First Author (Year)	Country	Study Design	Population	GA or BW	Number/case	ROP Diagnosis	Impact of HDP on ROP	Adjustments	Risk of bias
Huang (2016)^13^	China	Prospective/cohort	database of the Premature Baby Foundation of Taiwan	VLBW infant	5718 /844	Registatary data	Preeclampsia: any stage, OR = 1.00[0.84–1.20]; severe stage, OR = 0.89[0.83–1.25]	GA, BW, cesarean section, sex, Apgar score, transfusion, sepsis, distress syndrome with surfactant treatment, patent ductus arteriosus.	Only VLBL infants; NOS = 7
Gagliardi (2014)^9^	Italian	Prospective/cohort	82 hospitals adhering to the Italian Neonatal Network	GA<30w; BW<1501g	3606	Registatary data	HDP: severe stage, OR = 1.48[1.02–2.15]	GA, antenatal steroids, gender, multiple pregnancies, inborn/outborn, mode of delivery	Only VLBL infants; NOS = 7
Araz-Ersan (2013)^14^	Turkey	Retrospective/ cohort	Istanbul Faculty of Medicine in Turkey	Preterm infant	788	Medical record	Preeclampsia: severe stage, OR = 0.21[0.09–0.52]	Sepsis, Male gender, Multiple gestation, BW<1500	Only preterm infants; NOS = 7
Gagliardi (2013)^8^	Italian	Retrospective/cohort	the Accesso alle Cure e Terapie Intensive Ostetriche e Neonatali study in Italian	GA:23-31w	2058/89	Medical record	HPD: severe stage, OR = 2.0[1.0–4.0]	level of birth center, GA	Only preterm infants; NOS = 8
Yu (2012)^15^	US	Retrospective cohort	12 clinical centers across 9 American College of Obstetricians and Gynecologists US districts	GA:23-36w	25473/1053	Medical record	Preeclampsia: any stage, OR = 0.66[0.50–0.87]; Gestational hypertension: any stage, OR = 0.85[0.54–1.34]	GA, mode of delivery, number of fetuses, race, BMI at delivery, BW, gender, intraventricular hemorrhage, blood transfusion, and congenital anomalies	Only preterm infants; NOS = 8
Ozkan (2011)^16^	Turkey	Prospective cohort	NICU of Uludag University School of Medicine in Turkey	GA<32w	385/109	Medical record	Preeclampsia: any stage, OR = 1.78[1.66–1.90]	GA, BW, Duration of mechanical ventilation, Duration of total oxygen	Only VLBW infants, NICU participant; NOS = 8
Fortes (2011)^17^	Brazil	Prospective / cohort	Hospital de Clínicas de Porto Alegre	GA≤32w BW≤1500g	324/97	Medical record	Preeclampsia: any stage, OR = 0.41[0.20–0.82]; severe stage, OR = 0.20[0.04–0.94]	GA, Antenatal steroid treatment, Essential hypertension, Any grade of intraventricular hemorrhage, Use of oxygen in mechanical ventilation, Use of indomethacin, Blood transfusion, Vaginal delivery Small for gestational age	Only VLBW infants; NOS = 8
Mehmet (2011)^18^	Turkey	Retrospective/ cohort	NICU of Dr Behcet Uz Children'Hospital in Turkey	GA<37w	203/86	Medical record	Preeclampsia: any stage, OR = 1.41[0.58–3.43]	-	Only preterm infants; NOS = 7
Zayed (2010)^19^	US	Retrospective/ cohort	Hospital databases and charts of all preterm inborn infants at the University of North Carolina	GA≤37w	5143/323	Medical record	new-onset gestational hypertension: any stage, OR = 1.32[1.01–1.72]	-	Only preterm infants; NOS = 7
Yang (2010)^20^	China	prospective/ cohort	NICU of Chang Gung Children’s Hospital in Taiwan	VLBW infant	216/99	Medical record	Preeclampsia: any stage, OR = 2.52[1.32–4.7]; severe stage, OR = 0.39[0.05–3.29]	Duration of mechanical ventilation, BW	Only VLBW infants, NICU participant; NOS = 7
Shah (2005)^21^	Singapore	Retrospective/ cohort	The Neonatal Department of the Singapore General Hospital	VLBW infant	564/165	Medical record	Preeclampsia: any stage, OR = 2.51[1.32–4.7]	BW, Duration of CPAP, Duration of mechanical ventilation, Pulmonary haemorrhage	Only VLBW infants; NOS = 7
Seiberth (2000)^5^	Germany	Retrospective/ cohort	University Children’s Hospital in Heidelberg and Mannheim and Worms City Hospital in Germany	BW≤1500g	402/145	Registatary data	Preeclampsia: any stage, OR = 0.52[0.32–0.86]		Only VLBW infants; NOS = 6
Gerd (1996)^10^	Sweden	Retrospective/ cohort	population-based study in Sweden	BW≤ 1500g	202/81	Registatary data	Preeclampsia: any stage, OR = 0.69[0.37–1.30]		Only VLBW infants; NOS = 5

BW: birth weight; GA: gestational age; VLBW: very low birth weight; HDP: hypertensive disorder in pregnancy; mHTN: maternal new-onset gestational hypertension, NICU: neonatal intensive care unit; NOS: Newcastle-Ottawa scale scores.

### Quality assessment

The studies were heterogeneous and various sizes; nine studies included between 100 and 1000 participants, and three studies had more than 1000 participants, with a range from 202 to 25473. More than half of the studies were retrospective and recruited consecutive patients. Most studies provided data on diagnosis of ROP in the at-risk population, and they reported the mortality rate of patients with ROP. Five studies adjusted for potential confounding factors. All included publications scored >5 assessed by the NOS S1 Table.

### HDP and any stage of ROP

**Fig 2:**
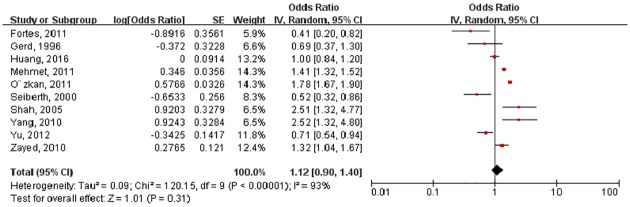
Forest plot showing the association between any stage retinopathy of prematurity and hypertensive disorders in pregnancy.

**Fig 3:**
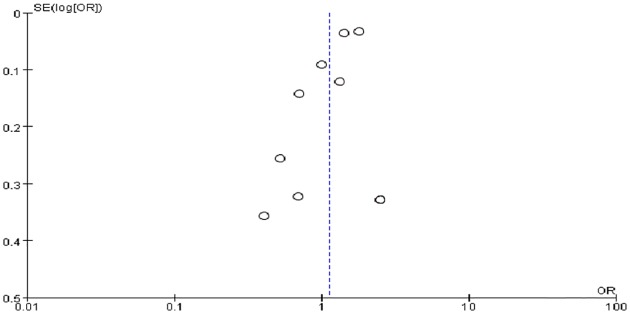
Funnel plot showing publication bias of any stage retinopathy of prematurity and hypertensive disorders in pregnancy.

Ten ORs of any stage ROP were pooled in the meta-analysis. However, the ORs for the association varied from 0.20 to 2.52 across studies. The pooled OR from the random-effects model was 1.12 (95%CI: 0.90–1.40) (Fig 2). Substantial heterogeneity existed in this estimate (P < 0.01; I^2^ = 93%). Sensitivity analyses confirmed that no single study qualitatively influenced the pooled OR. Visual inspection of the funnel plot identified no substantial asymmetry for HDP and any stage (Fig 3). The modified Bgger’s test showed no publication bias (P = 1.00).

### HDP and the severe stage of ROP

**Fig 4:**
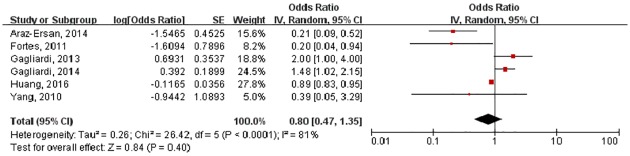
Forest plot showing the association between severe stage retinopathy of prematurity and hypertensive disorders in pregnancy.

Six studies reported OR of severe ROP and were pooled in the meta-analysis. Fig 4 shows the combined results of severe stage of ROP from the random-effects model. The pooled OR was 0.80 (95%CI: 0.47–1.35). Substantial heterogeneity was observed (P < 0.01; I^2^ = 81%). Sensitivity analyses confirmed that no single study qualitatively influenced the pooled OR. The modified Begg’s test confirmed that there was no publication bias (P = 0.624).

### Subgroup analysis

Stratified meta-analyses in subgroups according to study design (retrospective cohort or prospective cohort), OR style and HDP classification revealed similar results. The results showed that control for duration of mechanical ventilation (Coef. = -0.95, p-value = 0.021) was a significant predictor of ROP, but study design, HDP classification, and adjusted OR or not measurements were not (p>0.09) (Table 2).

**Table 2:** Subgroup analysis.

Subgroup	Number of risk estimate	Heterogeneity		Pooled OR	Adjusted R^2^
		Q	I^2^		
**Any stage of ROP**					
-Study design					-12.72%
retrospective	6	<0.001	89%	1.03[0.73 1.46]	
prospective	4	<0.001	94%	1.22[0.74 1.99]	
-Control for duration of mechanical ventilation					54.98%
yes	3	0.34	8%	1.84[1.59 2.12]	
no	7	<0.01	89%	1.03[0.78 1.36]	
-HDP classification					-17.41%
HDP	1			0.71[0.54 0.94]	
preeclampsia	8	<0.01	92%	1.18[0.93 1.50]	
Gestational hypertension	1	0.65		1.32[1.04 1.67]	
**Severe stage of ROP**					
-Study design					-39.79%
retrospective	2	<0.01	93%	0.66[0.07 5.96]	
prospective	4	0.01	73%	0.91[0.55 1.51	
-OR					-28.97%
Adjusted OR	1			0.39[0.05 3.29]	
Crude OR	5	<0.01	85%	0.82[0.48 1.43]	
-HDP classification					19.08%
HDP	2	0.45	0%	1.58[1.14 2.20]	
preeclampsia	4	0.03	79%	0.39[0.14 1.09]	
Gestational hypertension	0				

## Discussion

To our knowledge, this meta-analysis is the first of its kind that analyzes the effect of HDP on the development of ROP in infants. The meta-analysis of data extracted from included studies showed that the OR of developing ROP was no different between infants exposed to maternal hypertension and those who were not. Even though there seems to be an increased ROP rates in infants with exposure to maternal HDP in some studies [8–9], this has not been supported by findings across all studies. In the subgroup analyses, results and heterogeneity were similar to those of the pooled analysis. An odds ratio of 1.12 was found between HDP and ROP, but wide divergence in study results contributed to our confidence interval crossing unity. The non-significant association between AMD and stroke may arise from several sources.

The incomplete or various possible confounding factors not adjusted in studies may have influenced the results. Of the thirteen studies included, nine attempted to control for gestational age or birth weight of infants [8–9,13–14,16–19,21]; and three studies that adjusted for the duration of mechanical ventilation supported preeclampsia as a risk factor for developing ROP in offspring [14,17,19]. Few studies adjusted for the presence of antenatal use of steroids and oxygen. None of the included studies examined the role that other pregnancy disorders might have in played in influencing the association between HDP and ROP. Most studies recruited infants of < 1500 g birth weight, and some were from NICU with hospital control. It is possible that the meta-analysis results are representative of low birth weight subjects. In addition, few studies are focused on this topic, This caused the heterogeneity and the skewing of results when one study dominated in the weighting.

Although we found no overall differences in HDP, some category-specific differences were observed between infant exposure to preeclampsia and gestational hypertension. Yu et al investigated the impact of maternal gestational hypertension and preeclampsia on the occurrence of ROP in preterm infants and reported that preeclampsia, but not gestational hypertension, was associated with a significantly reduced risk of ROP [15]. It is well recognized that maternal HDP leads to low birth weight and small-for-gestational-age infants, which are important risk factors for ROP [16]. However, the potential protective effect on ROP cannot be ruled out.

As we know, overproduction of VEGF is an important mechanism in physiologic retinal vascular development and pathologic angiogenesis in the preterm infant retina [22]. sFlt1, a specific endogenous inhibitor of VEGF, can combine with VEGF and prevent it from mediating its biological activities through its receptors [23]. Using a murine oxygen-induced ischemic retinopathy (OIR) model, mice treated with lenti.sFlt-1 demonstrated a more marked reduction in neovascularization in their retinas than untreated mice [24]. Furthermore, treatment with intravitreal bevacizumab, an anti-VEGF agent, for retinopathy of prematurity resulted in regression of neovascularization [25]. Interestingly, elevated levels of antiangiogenic factors, such as sFlt-1 and PlGF, were detected in preeclampsia [26–27]. Recent studies demonstrated that serum levels of circulating sFlt1 and PIGF changed as HDP developed. Preeclampsia patients showed higher sFlt1 and lower PIGF levels than patients with pregnancy-induced-hypertension and gestational proteinuria. This change of angiogenic level was also evident in varying degrees of severity of preeclampsia [28–29]. The elevated antiangiogenic factors that are produced by the placenta and enter into the systemic circulation may also have effects in the fetus.

On the basis of our findings, several questions arise. First, is the real association between HDP and ROP. Because of limited studies and variable study quality, the results of meta-analysis must be interpreted with caution, and the current article could not provide enough evidence to determine the relationship between HDP and ROP. Further studies should be considered and should include more representative participants, account for the different types of HDP, and adequately control for confounding factors. Second, if HDP increases the risk of ROP, can we treat HDP through drug intervention, lifestyle modification, and/or dietary therapy to protect against ROP? Third, the exact mechanisms underlying the relationship between HDP and ROP have yet to be elucidated. More studies are needed to increase understanding of this association and to provide convincing evidence for ROP prevention.

## Supporting information

S1 Table The Newcastle-Ottawa scale score of included studies. (DOC)

S2 Table PRISMA 2009 checklist. (DOC)
