# Supplementary material for: Association of maternal hypertensive disorders with retinopathy of prematurity: A systematic review and meta-analysis
Source: PLoS One. 2017 Apr 7;12(4):e0175374. doi: 10.1371/journal.pone.0175374 (PMC5384774; doi:10.1371/journal.pone.0175374)
Supplement: S1 Table — (DOC) [file pone.0175374.s001.doc]

| STable 1. The Newcastle-Ottawa Scale score of included studies | | | | | | | | |  |
| --- | --- | --- | --- | --- | --- | --- | --- | --- | --- |
| study | Selection of exposured | Selection of non-exposed | assess of exposure | outcome | Comparability | Assess of outcome | long enough of follow up | Adequacy of follow up | |
| Huang (2016) | 1 | 1 | 1 | 1 | 1 | 0 | 1 | 1 | |
| Gagliardi (2014) | 1 | 1 | 1 | 1 | 1 | 0 | 1 | 1 | |
| Ozkan  (2011) | 1 | 1 | 1 | 1 | 1 | 1 | 1 | 1 | |
| Gagliardi  (2013) | 1 | 1 | 1 | 1 | 1 | 1 | 1 | 1 | |
| Zayed  (2010) | 1 | 1 | 1 | 1 | 0 | 1 | 1 | 1 | |
| Yang  (2010) | 0 | 1 | 1 | 1 | 1 | 1 | 1 | 1 | |
| Bilge  (2013) | 0 | 1 | 1 | 1 | 1 | 1 | 1 | 1 | |
| Shah  (2005) | 0 | 1 | 1 | 1 | 1 | 1 | 1 | 1 | |
| Filho  (2011) | 1 | 1 | 1 | 1 | 1 | 1 | 1 | 1 | |
| Mehmet  (2011) | 1 | 1 | 1 | 1 | 0 | 1 | 1 | 1 | |
| Yu  (2012) | 1 | 1 | 1 | 1 | 1 | 1 | 1 | 1 | |
| Seiberth  (2000) | 1 | 1 | 1 | 1 | 0 | 0 | 1 | 1 | |
| Gerd  (1996) | 0 | 1 | 1 | 1 | 0 | 0 | 1 | 1 | |
